# Emergency fueling unit for means of heavy transport affected by electromagnetic pulse

**DOI:** 10.1038/s41598-022-06087-w

**Published:** 2022-02-08

**Authors:** Grzegorz Pawlak, Patryk Płochocki, Przemysław Simiński, Tomasz Skrzek

**Affiliations:** 1 Military Institute of Armored and Automotive Technology, Okuniewska 1 Street, 05-070 Sulejówek, Poland; 2 Kazimierz Pulaski University of Technology and Humanities in Radom, Faculty of Mechanical Engineering, Chrobrego 45 Street, 26-600 Radom, Poland

**Keywords:** Magnetospheric physics, Electrical and electronic engineering, Mechanical engineering

## Abstract

The electromagnetic pulse (EMP) is either a natural phenomenon caused by a solar storm or can be an effect of the deliberately use of destructive weapons. It is a threat to critical infrastructure systems, including transportation. The construction of transportation means continues to pursue the rapidly accelerating economy and ecological demands while increasingly becoming more dependent on electricity and electronics each day. The paper presents a method that enables driving a vehicle after an impact of EMP of high energy, where the standard protection systems like insulations or protection shields are not efficient, and the vehicle electrical system is destroyed. The device called the Emergency Fueling Unit (EFU) is described. EFU was installed and tested on a truck equipped with a six-cylinder, 11 L CI engine. The truck acceleration test results presented in the paper confirm that the EFU could be effectively used to drive the truck in case of the destruction of its electrical system. The paper’s topic also provides an opportunity to reflect on how and why modern vehicles' powertrains, especially commercial vehicles, are exposed to the influence of phenomena that do not occur in everyday life but can have a disastrous impact on means of transportation and our safety.

## Introduction

In 1859, an electromagnetic pulse emitted during a solar storm caused damage to the man-made infrastructure on a large scale. It struck the Earth and ruined most telegraph systems in operation in 1859. The phenomenon is known as the Carrington Event^1^. It was the first time people experienced a real threat to electrical installations that had just begun to emerge. In 1921 a geomagnetic storm called the Railroad Storm struck North America. At that time, the electrical grid was still underdeveloped, and there were no catastrophic consequences.

Now, it would result in a devastating power cut lasting 4–10 years and costing trillions of dollars^2^. In^3^ Pete Riley defines the term “extreme” as: “That is, an event that we have not yet observed during the time our society has become dependent on technology, and which could result in significant adverse consequences affecting a significant fraction of the Earth's population”. Now, we know a lot about EMP phenomena, especially its natural sources and consequences for our society. Still, we do not know when such an extreme phenomenon will happen next time, and we cannot predict its intensity. According to Pete Riley the probability of another Carrington event occurring within the next decade could be as high as 12%. Love in^4^ assumes that the probability of extreme geomagnetic storms is 6% per decade. In^5^, R. Kataoka writes: “It is found that the probability of occurrence of extreme magnetic storms can be modelled as a function of maximum sunspot number of a solar cycle, and the probability of another Carrington storm occurring within the next decade is estimated to be 4–6%”—still, a lot. Authors of^6^ conclude that a magnetic storm with intensity exceeding that of the 1859 Carrington event occurs about 1.13 times per century and has a wide 95% confidence interval of [0.42, 2.41] times per century. Out of the ten most giant storms^7^, the two most significant were observed in 1989, March 14 and 2003, October 29. The first one sent Quebec into darkness (knocked out power) for 9 h.

The EMP destructive effect is utilised in the deadliest and terrifying weapons, causing local or massive infrastructural damage. A nuclear weapon detonated above the Earth produces an EMP that is a source of catastrophic current and voltage surges to electrical/electronic equipment and systems. There are three nuclear EMP components (E1–E3). The first (E1) appears when gamma radiation from the atomic detonation ionises atoms in the upper atmosphere. The E1 pulse may reach peaks of about 50,000 V/m. In contrast, the vehicle producer's harmonised immunity standards for vehicle's components foresee testing them for 100–200 V/m. Another possible deadly threat to transport is directed-energy weapons (DEW). The DEW can generate an electromagnetic field with an intensity of up to 300 kV/m^8^. An example of a new generation weapon is high power microwave (HPM) pulses. As a mass destruction weapon, it does not kill, is not selective, acts on the attackers and attacked, and there is no possibility to avoid the consequences of its application. HPM pulses are the best example to show the weakness of the protections of electronic systems. They could generate very high currents on structures exposed to them. It disrupts the software of most of the currently used semiconductor systems. Same fibre-optic cables are immune, but fibre optic connectors and fibre optic systems with attached converters are the most susceptible elements to HPM exposure^9^. There are many materials in the form of shields used to protect electronic units against magnetic waves. The materials' absorption characteristics depend on the angle of incidence and magnetic waves frequency^10^. Their protection capability is limited to a pulse's specific power. In the case of electronic equipment protection, the Faraday Cage is efficient, but it is rarely used in civil transport due to extra costs.

Human activity and creativity seem to be less predictable than natural phenomena, so we cannot foresee when, where and how the destructive capabilities of EMP can be used. But we can assume that it will happen. There are no public results that illustrate the scale of the danger posed by the possible impact of EMP on modern vehicles and the transportation system. However, the results of tests of EMP influence on older vehicles are the cause for concern^11^.

Now, we rely on vehicles for everything, from supplying goods, rubbish collecting to faraway trips. But our modern vehicles, especially commercial vehicles, are generally not EMP-proof. If they were to stop, even getting out of town would not be possible for most people.

The paper describes the work results devoted to finding a solution enabling starting the vehicle and driving it after a severe EMP impact when the conventional EMP protection systems failed and the electrical installation is destroyed. Because IC engines power most commercial vehicles, the described research focused on heavy trucks and CI engines. The emergency fueling unit (EFU) is an add-on system dedicated to CI engines, especially for heavy vehicles in emergencies. It allows the engine to run without a standard fuel system. The unit enables the air–fuel mixture preparation and effective combustion of the fuel to ensure the engine power is high enough to drive a loaded vehicle. The solution excludes electrical and electronic components and does not interfere with the vehicle's structure.

## The role of the IC engine in transport

97.8% of all trucks in the EU run on diesel oil, while petrol fuels 1.3% of the fleet. 0.04% of trucks on the EU roads are zero-emissions^12^. By 2030, the overall adoption of new-energy vehicles for light commercial vehicles across key markets will exceed 35%, and heavy-duty trucks will be around 26%. It means that the transportation system is based on a CI engine, and the situation will not change rapidly in a decade. But in the next decade (up to 2030), IC engines will be more efficient. Thermal and mechanical losses will drop by more than 22% (compared to the average European truck from 2015)^13^, so the lower fuel consumption could make the CI engine still a competitive solution. There is no doubt that Euro 7 standards will strongly influence powertrain development. According to preliminary data, CO_2_ emissions should be reduced by 66% compared to Euro 6D, nitrogen emissions by half, and particulate matter emissions by six times. The new Euro 7 standards will contribute to a modal shift from car-based cities to walking, cycling, and public transport**,** wherever possible. The reason is the production of many smaller, more affordable IC engine cars would be economically unviable, or the price would be high. Association for Emission Control by Catalyst, in its AECC position paper, inform: “Emissions fluctuate in real-world operation depending on the driving conditions, e.g. due to the impact of the ambient condition, the trip composition or the driving dynamics. Euro 7/VII should ensure that emission control systems are appropriate to handle such variation in real-world emissions. AECC acknowledges that imposing the same requirements up to the most extreme driving conditions might not be the best cost/benefit scenario but legislation must cover all conditions commonly experienced in populated areas to be effective, including road gradient changes, real acceleration rates and vehicle loadings”^14^.

Consequently, the IC engine's role will be minimised or eliminated from urban transport. Still, an efficient IC engine in commercial transportation has a much better perspective than announced for more than two decades. So, now and for the foreseeable future, the CI engine will secure goods transport, and its reliability is a base of the functioning of transportation. New emission standards make engines and their control systems even more complex and more vulnerable to EMP. Unfortunately, the issue of securing the functioning of modern means of transport in extreme situations is not sufficiently developed. Considering that the IC engine is still the primary power unit for transportation, it is worth looking at the possibility of its functioning in an emergency when the electronic units and other critical components do not function.

## Mixture creation and combustion process in CI engine

Engine control systems enabling the combustion process control allow the fulfilment of emission standards but make the vehicle more vulnerable. When attempting to develop a system that allows the engine to start and operate in conditions of damage to electronic systems and electrical components, one should consider the processes of mixture formation and combustion, which are the most important from the point of view of engine operation. First of all, it is necessary to analyse the engine control unit (ECU) functions so that at least in part, they could be replaced by the emergency fueling with engine control elements.

**Figure 1:**
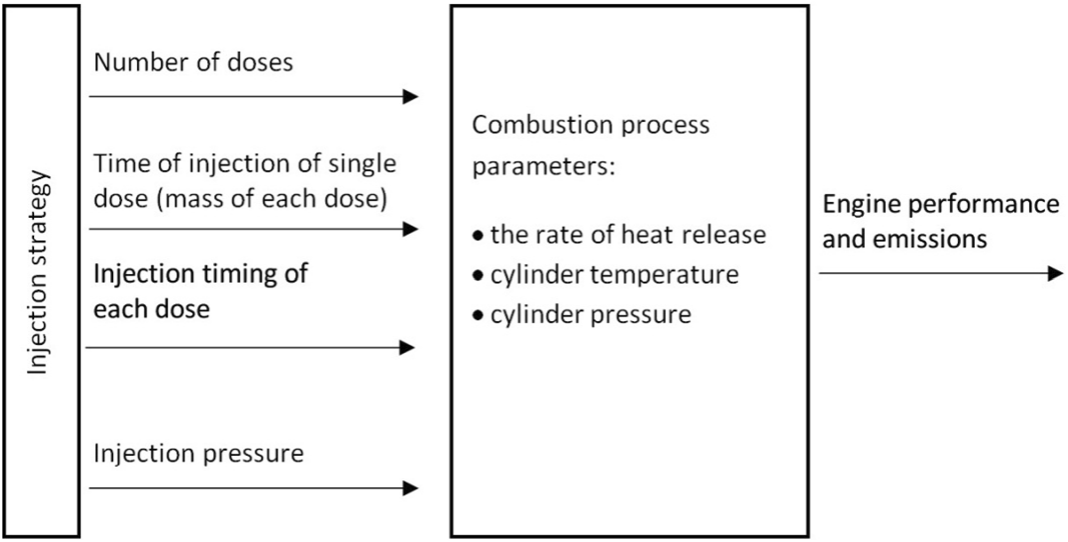
The control of combustion realised by the Engine Control Unit (ECU). (Source: Authors).

The combustion process organisation in the CI engine determines the engine emissions and their efficiency. Despite the development of a homogeneous charge compression ignition (HCCI) process, which is very demanding in terms of combustion control^15,16^ the CI engine's dominant strategy is Partial Premixed Combustion (PPC)^17–19^. Fuel is directly injected into the combustion chamber in PPC mode, and a stratified premixed charge is burned in a controlled way (Fig. 1).

For the process efficiency, the air–fuel mixture creation is crucial. The realisation of injection strategies enables advanced fuel systems like common rail (CR) with sophisticated, electronic injection process control. The PPC heat release process includes four phases:

1. ignition delay,
2. low-temperature reaction,
3. premixed combustion phase,
4. late mixing controlled phase.

The initial fuel dose (doses) injection several degrees before top dead centre (TDC) contributes to radicals in the combustion chamber. The fuel's main dose is injected close to the TDC and could be burnt efficiently because of an active space created by initially injected fuel and EGR mixed with compressed air. Controlled combustion is also possible because of the size structure of injected droplets. The shape of the stream of evaporating droplets is also essential. The experimental results described in^20,21^ indicate that reducing nozzle cone angle, increasing injection pulse number, and advancing the injection timing are effective ways for nitrogen oxides emission reduction and lowering the hydrocarbons and carbon monoxide emissions level. The physical properties of fuel have a profound influence on the nozzle flow and spray characteristics that were explained in^22^ with the use of different fuels. The size of the droplets is controlled by injection pressure. In the new fuel systems, it could be more than 3000 bars.

The simplified mixture creation and combustion process description show what could happen when the injection process's proper control is impossible. It happens the engine electronic control unit (ECU) or any vehicle CAN element necessary for the engine control is damaged or destroyed. Even if the ECU operates correctly, but incorrect signals from the sensors appear, engine performance may be reduced, or the engine could be stopped^8^. The engine stop may also be caused by faulty action of the control unit (e.g. disruption of the internal processor).

## Methodology

The research problem was an elaboration of an effective method that allows starting and driving a truck equipped with a modern CI engine, with electrical and electronic systems, including the ECU destroyed by EMP. It was assumed that such a damaged vehicle was to drive on a paved road and off-road for a distance of at least 5 km, loaded at least 60% of the permissible load. The minimum speed was not assumed while at the same time trying to make it as high as possible. The vehicle after an emergency ride should be operational after replacing and repairing the elements and systems damaged. Because it was assumed that using the vehicle standard parts and units after destruction would be impossible, an autonomous emergency fueling unit (EFU) is necessary.

One of the biggest problems to be solved was the proper air–fuel mixture formation process and the realisation of the combustion process without the engine knocking or misfiring. It required a non-standard method of creating an air–fuel mixture and choosing a proper fuel. Also, pneumatic installation on the vehicle is required to provide energy for a planned fueling unit.

This part of the work required the laboratory tests described in point 5. The tests included using a one-cylinder research engine AVL 5402, to check the possibility of controlling the combustion process of the air–fuel mixture formed outside the engine cylinder. The results of preliminary tests allowed the construction of the full-size EFU. The first laboratory tests of EFU were carried out on the test stand with Scania DC9 engine (a 9-L, five-cylinder engine) and Schenck Dynabar engine brake. The single heads per cylinder facilitated the EFU test process, especially in the modification phase of its construction. In the initial phase of work, only one full-size engine cylinder was fueled unconventionally, which enabled adjusting its working parameters. The final laboratory test with the use of Scania DC9 engine gave the possibility to check the engine performance and its control capability. Scania engines have a modular construction, so the experience and results obtained for the DC9 engine were easily applied to DC11 (11-L, six cylinders engine) from the Scania P340 truck. The truck was used for practical verification of EFU capabilities. The truck parameters, especially its speed and acceleration, were measured and registered by the vehicle dynamics testing system (OXTS RT3002). The truck tests were supposed to allow not only to verify the assumptions related to the vehicle's achievement of operating parameters but also to give the possibility of a practical test of the possibility of operating the EFU and controlling the vehicle by the driver, which was a crucial element of the project.

## The idea of the EFU and its experimental verification

There are no solutions to enable the start-up and operation of a modern CI engine with a damaged or destroyed engine control system and its electrical installation. One of the consequences of the destruction of vehicle electrical installation, particularly ECU damage, is the blockade of fuel injectors. As a result, the complex mixture creation and combustion control processes outlined in p. 3 (Fig. 1) cannot be realised. Initially, a thickening of the cylinder head gasket and the introduction of fuel channels there was considered, but it could affect its regular operation and would reduce the reliability of the engine, so the idea was rejected and continuous, indirect fuel injection was proposed. The continuous injection of the fuel to engine inlet channels had been utilised in old generation SI engines (e.g., Bosch K-Jetronic fuel system)^23^. Also, in dual-fuel CI engines, low reactive liquid fuels like methanol^24^, ethanol^25^ or gasoline^26^ are provided to the inlet manifold or inlet channels. The air–fuel homogeneous mixture created this way can be ignited by a pilot dose of highly reactive fuel (e.g. diesel fuel, vegetable oils or their esters), injected into the engine cylinder^27^.

In the proposed method of emergency CI engine fueling, a highly reactive fuel was planned to be used. The combustion of such a fuel supplied to the inlet channel demands the preparation of well-mixed charge due to its lower volatility. So, the external arrangement is required to vaporise it^28^. External charge preparation using diesel has been explored using air-assisted injectors and external diesel vaporisers. In the air-assisted injection system, fuel is injected using a gasoline port injector inside an air-assisted cap, where it mixes with the externally supplied air^29^. In other solutions, fuel is injected into the externally heated chamber in an external diesel vaporiser, creating diesel vapours^30,31^. In the process, the homogeneous air–fuel mixture is created. Combustion of it carries a risk of uncontrolled self-ignition, usually starts very early and is relatively very fast. In^30^, the ignition delay was extended by retarding the injection timing, but the continuous injection must be applied in the proposed EFU. Also, an additional air throttle was excluded to simplify its construction. The amount of injected fuel was the only way to change the mixture composition and become the only parameter for control of the combustion and, as a result, engine output. Such conditions demanded a unique way of mixture creation connected with fuel atomisation. The fumigation of the injected fuel with the air in an especially constructed injector was proposed.

**Figure 2:**
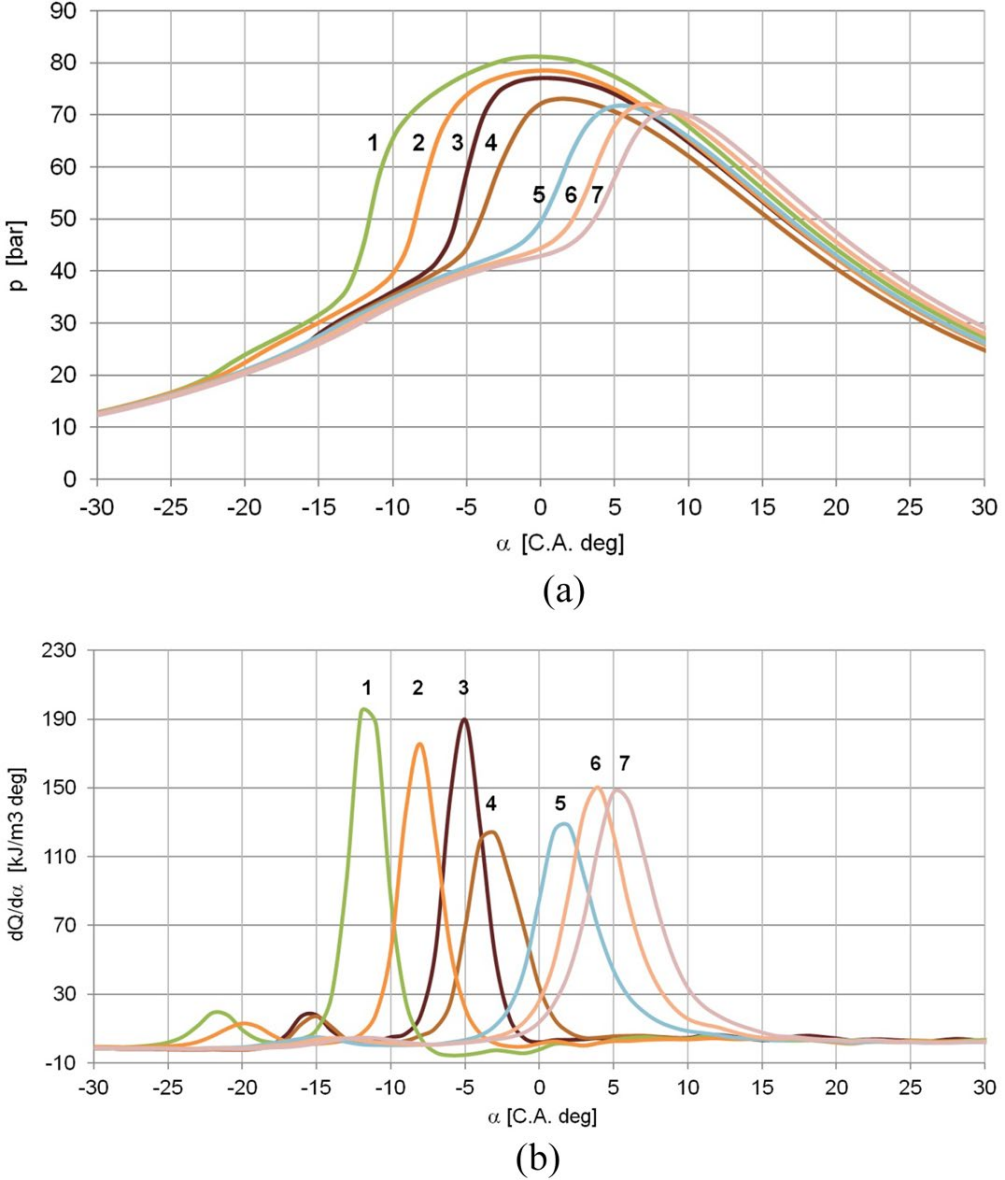
Cylinder pressure diagrams (**a**), and the rate of heat release (**b**) for engine fueling with JP-8 without mixture dilution (curves no. 1, 2, 3, 4) and with dilution using CO_2_ (curves no. 5, 6, 7) for the mixture created outside engine cylinder, n = 1200 rpm. Curve number: 1—JP-8 (λ = 1.76, BMEP = 2.5 bar, η_th_ = 22.2%, dp/dα = 12.6 bar/deg), 2—JP-8 (λ = 2.00, BMEP = 2.3 bar, η_th_ = 23.4%, dp/dα = 10.9 bar/deg), 3—JP-8 (λ = 2.04, BMEP = 2.8 bar, η_th_ = 38.1%, dp/dα = 11.8 bar/deg), 4—JP-8 (λ = 2.14, BMEP = 2.5 bar, η_th_ = 39.1%, dp/dα = 8.0 bar/deg), 5—JP-8, 10% CO_2_ (λ = 1.85, BMEP = 2.8 bar, η_th_ = 24.1%, dp/dα = 7.3 bar/deg), 6—JP-8, 15% CO_2_ (λ = 1.46, BMEP = 3.6 bar, η_th_ = 21.0%, dp/dα = 7.8 bar/deg), 7—JP-8, 20% CO_2_ (λ = 1.26, BMEP = 4.1 bar, η_th_ = 19.8%, dp/dα = 7.5 bar/deg). (Source: Authors).

Examination of the combustion process of the air–fuel mixture created outside the engine cylinder started with using one-cylinder research engine AVL 5402 (compression ratio CR = 17) applying gasoline port injector. The engine was fueled with diesel oil. The test was not successful. The self-ignition of diesel oil in the combustion chamber was not controlled sufficiently, even for small diesel oil doses. Then JP-8 (NATO code F-34—the highly reactive fuel used to replace diesel oil in military applications) was tested. Its combustion process strongly depended on the excess air coefficient (λ) and generally, even for small doses of the fuel (λ > 2), the self-ignition was too early (Fig. 2). The phenomenon was also observed in the experiment carried out on the engine with a low compression ratio (CR = 12) fueled with homogeneous mixtures of JP-8^16^. For leaner mixtures, the combustion process was retarded, but the high rate of cylinder pressure rise (dp/dα) was not acceptable. Curves 1, 2, 3, 4 in Fig. 2a,b showed the cylinder pressure (p) diagrams and volumetric heat release rate (dQ/dα) for JP-8 fueling when the dp/dα was limited to 8 bars/deg. The higher values of dp/dα resulted in the engine knock. The combustion of JP-8/air mixtures created outside the engine cylinder did not allow brake mean effective pressure (BMEP) higher than 2.8 bar. The controlled delay of self-ignition enabled dilution of JP-8/air mixture using CO_2_ (curves 5, 6, 7 in Fig. 2). The method gave higher BMEP, but from a practical point of view (additional gas tank and extra control elements in EFU), this method of controlled delay of self-ignition of the air–fuel mixture was rejected. This part of the experiment showed that it is necessary to find a more straightforward solution for the controlled self-ignition delay of the air–fuel mixture.

The self-ignition of the air–fuel mixture depends on fuel droplets evaporation. For the engine conditions, evaporation takes place when the temperature of the surroundings of the fuel drop is higher than the boiling point. Only part of the droplets evaporate before ignition, and the more significant amount evaporates after ignition in the diffusion-controlled phase of combustion^32^.

So, to realise the delayed combustion, it was necessary to slow the diffusion process. It was possible with the use of rapeseed oil mixed with JP-8 fuel. The fueling of one-cylinder research engine AVL 5402 with a blend of JP-8 and rapeseed oil allowed controlled self-ignition delay. This vegetable oil's density and kinematic viscosity are much higher than for JP-8 (Table 1). The significant share of the rapeseed oil in the blend (minimum about 30%) retarded self-ignition of the air–fuel mixture and enabled the engine to run with a limited knock or without it.

**Table 1:** The comparison of JP-8, rapeseed oil and diesel oil properties (Source: Authors, data from^33–35^).

Properties	Unit	JP-8	Rapeseed oil	Diesel oil
Low heating value	MJ/kg	42.8	37.1	43.2
Cetane number	–	45	48	50
Liquid density at 15 °C	kg/m^3^	804	920	831
Kinematic viscosity at 313 K	mm^2^/s	1.27	35.5	2.35
Carbon	kg/kg	0.857	0.774	0.862
Hydrogen	kg/kg	0.142	0.117	0.135
Oxygen	kg/kg	–	0.109	–
Flash point	°C	57	285	66
**Distillation**				
Start (initial boiling point)	°C	167	225	178
50%	°C	202	350	255
End	°C	238	380	353

The increased self-ignition delay of the JP-8/rapeseed oil blend can be explained by the higher temperature of the initial boiling point of rapeseed oil compared to JP-8. The higher share of rapeseed oil in the fuel blend increases the percentage of the fuel that burns under diffusion conditions, which lengthens the combustion process and reduces the engine knock. A significant advantage of using a blend of JP-8 with rapeseed oil is a possible high loading of the engine. The blend composition can be adjusted to the specific engine and its compression ratio, making the solution flexible. In addition, rapeseed oil is widely available, which is also essential considering that the system is intended for use in circumstances related to confusion and chaos.

**Figure 3:**
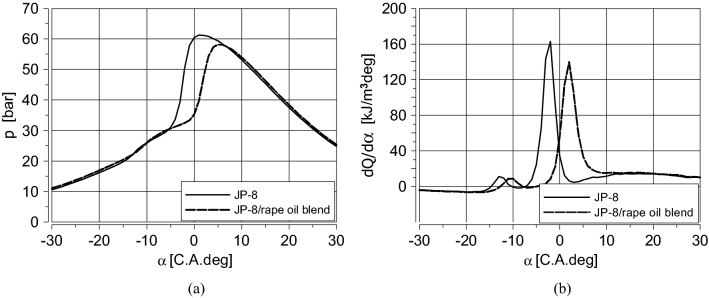
Cylinder pressure diagrams (**a**), and the volumetric heat release rate (**b**) for the same BMEP = 3.45 bar for fueling with JP-8 (η_th_ = 37.5%), and the blend of 70% of JP-8 and 30% of rapeseed oil (η_th_ = 22.9%), n = 1200 rpm. (Source: Authors).

Figure 3 presents the effect of fueling the engine with the blend of JP-8 with rapeseed oil (70% of JP-8 and 30% of rapeseed oil). The addition of rapeseed oil retarded the self-ignition of the mixture, so the heat release process was shifted by five crank angle degrees (Fig. 3b) and, as a result, maximum cylinder pressure occurred five degrees after TDC and was lower (Fig. 3a).

**Figure 4:**
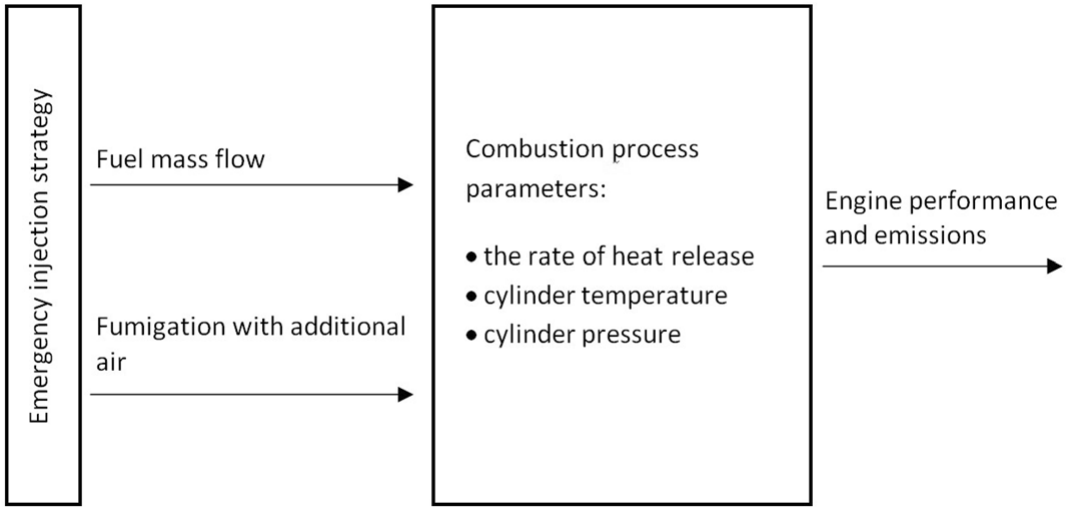
The control of combustion in the proposed fueling method. (Source: Authors).

The use of the JP-8/rape oil blend resulted in incomplete combustion, which affected the engine thermal efficiency (η_th_) but enabled to realise the proposed idea of air/fuel mixture creation outside the engine cylinder and partial control of its combustion (Fig. 4).

**Figure 5:**
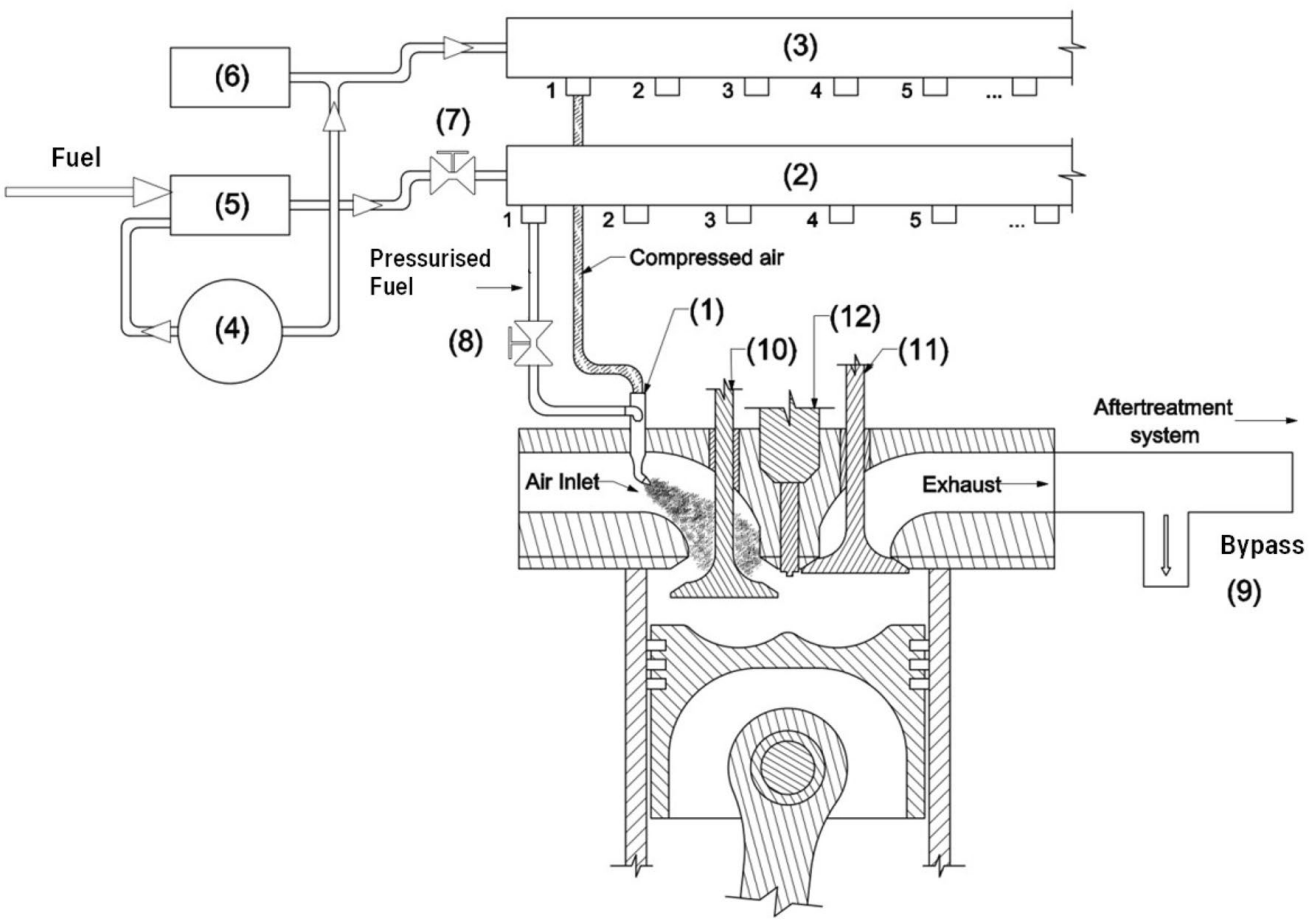
The idea of Emergency Fueling Unit (EFU). 1. stream injector with fuel fumigation, 2. fuel collector, 3. air collector, 4. air compressor, 5. pressurised fuel tank, 6. auxiliary air reservoir, 7. main fuel valve, 8. shut-off valve, 9. bypass, 10. intake valve, 11. exhaust valve, 12. the injector. (Source: Authors).

The experiment on fueling the engine with different fuels provided to the engine inlet channel has allowed the designing and construction of the EFU (Fig. 5).

The EFU is entirely a mechanical construction (Fig. 5). The fuel (JP-8/rapeseed oil blend) is stored in the tank (5) where the air is supplied under pressure. The air is provided to the fuel tank from the auxiliary air reservoir (6). After starting the engine, the truck air compressor (4) supplies the air to the auxiliary air reservoir. The pressurised air pushes fuel to the fuel collector (2) and then to the injectors (1). The outlets of injectors are located in the individual intake channels. The pressurised air from the auxiliary air reservoir is also supplied to the air collector (3) to fumigate the fuel stream in injectors. To atomise the fuel, about 4 bars of the air pressure was applied. The fuel provided to the injectors is controlled by the main fuel valve (7). The fumigated, rich mixture is supplied to the inlet channel under the inlet valves and, after their opening, to the combustion chamber.

For the laboratory test of the full-size engine, the EFU was installed on Scania DC9 engine. The engine was loaded up to 750 Nm for 1200–1600 rpm (maximum engine torque is 1250 Nm). For this engine power (P = 125 kW), the specific fuel consumption was about 520 g/kWh. One reason for high specific fuel consumption is that a part of the mixture was lost via exhaust valves during the overlap phase and was not completely burnt. The engine laboratory tests show that it is necessary to protect the aftertreatmet system, so the bypass unit for exhaust gases and unburned fuel was designed. For too lean air–fuel mixtures, the lack of self-ignition was observed, the idle run and low engine load were obtained by switching off the fuel for two cylinders (the EFU construction enables shutting off the fuel supply to each engine cylinder).

**Figure 6:**
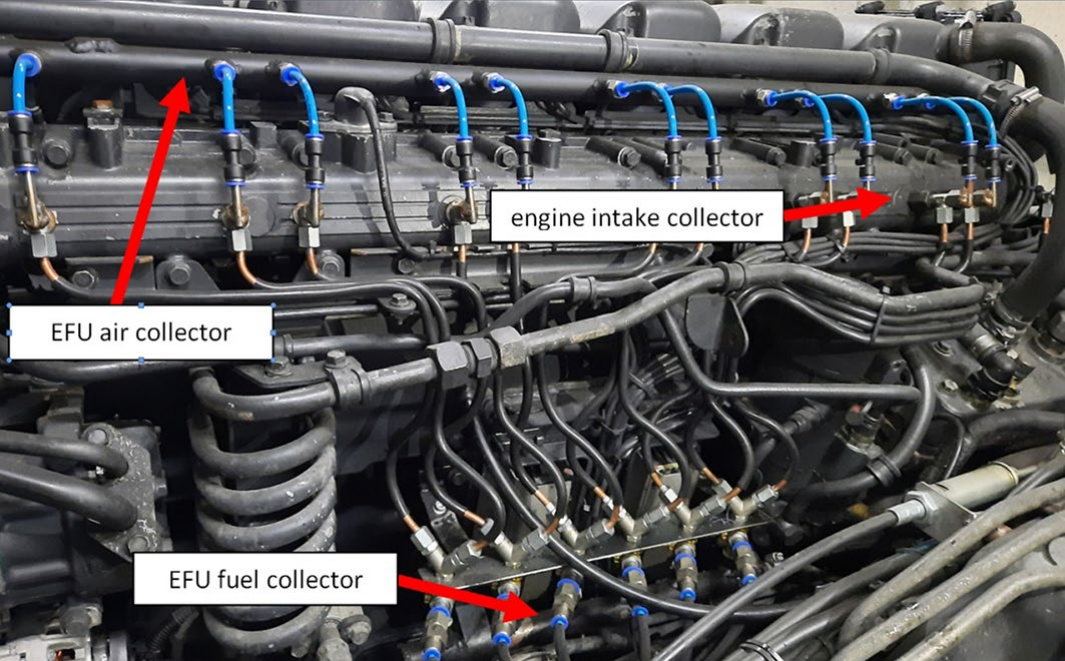
The DC11 engine with the EFU in the experimental truck. (Source: Authors).

The EFU operation was tested on the Scania P340 truck (Table 2). The main fuel valve of EFU was integrated with the truck acceleration pedal to control the engine load. The fuel cut-off valves allow switching off three out of six engine cylinders from work, ensuring a stable idle engine run and the run with low load. An inertial regulator united with the main fuel valve limits the maximum engine speed (about 2000 rpm). Figure 6 shows the Scania DC11 engine equipped with EFU.

**Table 2:** The experimental truck specification (Source: Authors).

Type	P340 DB6 × 2*4HNA
Gross vehicle weight (GVW)	26,000 kg
Axle distance	4700 mm
Engine	DC11 08/340HP (Euro 3)
Fuel system	Pumpinjectors (PDE)
Gearbox	GRS890R (manual)
Tyres	385/55 R22.5 (front axle) 315/70 R22.5 (rear axle)
Superstructure	Aluminium box with a crane
Empty truck weight	13,360 kg
Maximum load	12,640 kg
Loaded truck weight in the experiment	20,360 kg

## The practical verification of the EFU application

The test of the truck equipped with EFU consisted of the engine's start, the truck's acceleration on the concrete road (Table 3) and the drive on the distance of 5 km (50% ground and 50% concrete). The empty and loaded truck was tested. The load consisted of two concrete blocks, 3.5 tons each. The electric starter was used to start the engine, but a pneumatic starter should be installed in a version fully independent of the electrical system.

**Table 3:** Test road parameters for the truck acceleration tests (Source: Authors).

Parameter	Unit	Value
Type of surface	[–]	concrete
Test road length	[m]	180 ± 5
The slope of the test road to the longitudinal axis of the test vehicle	[deg]	0 ± 0.2
The slope of the test road to the transverse axis of the vehicle	[deg]	1.55 ± 0.5

The results show the comparison between the velocity and acceleration of the truck powered by the standard engine (fueled with diesel oil injected by pumpinjectors) and with the use of EFU (fueled with JP-8/rapeseed oil blend). Because the destruction of electric vehicle installation can make the gear changing impossible, only one gear (the third) was engaged, and it was not changed for the whole time for the standard fuel system and EFU fueling.

**Figure 7:**
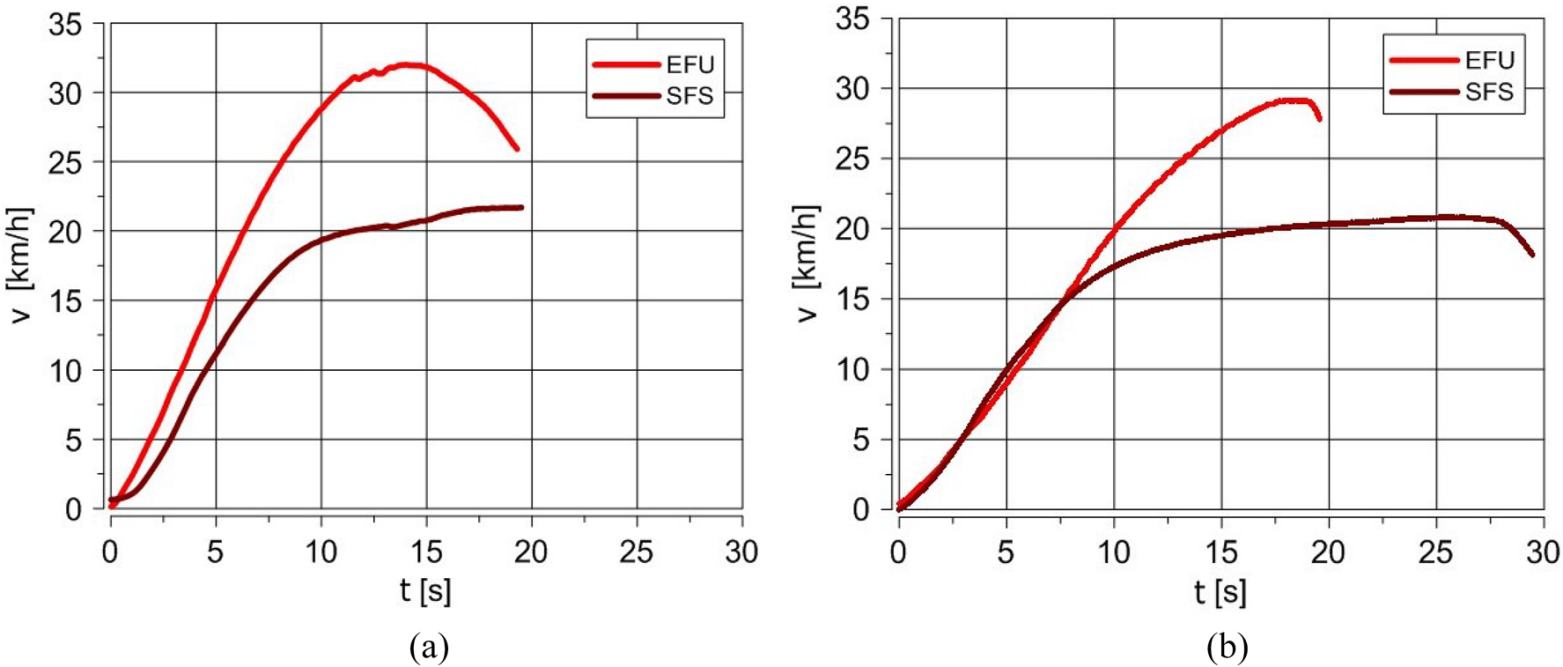
The comparison of truck velocity for standard and EFU fueling for an empty (**a**) and loaded truck (**b**) (EFU—Emergency Fueling Unit, SFS—Standard Fuel System). (Source: Authors).

**Figure 8:**
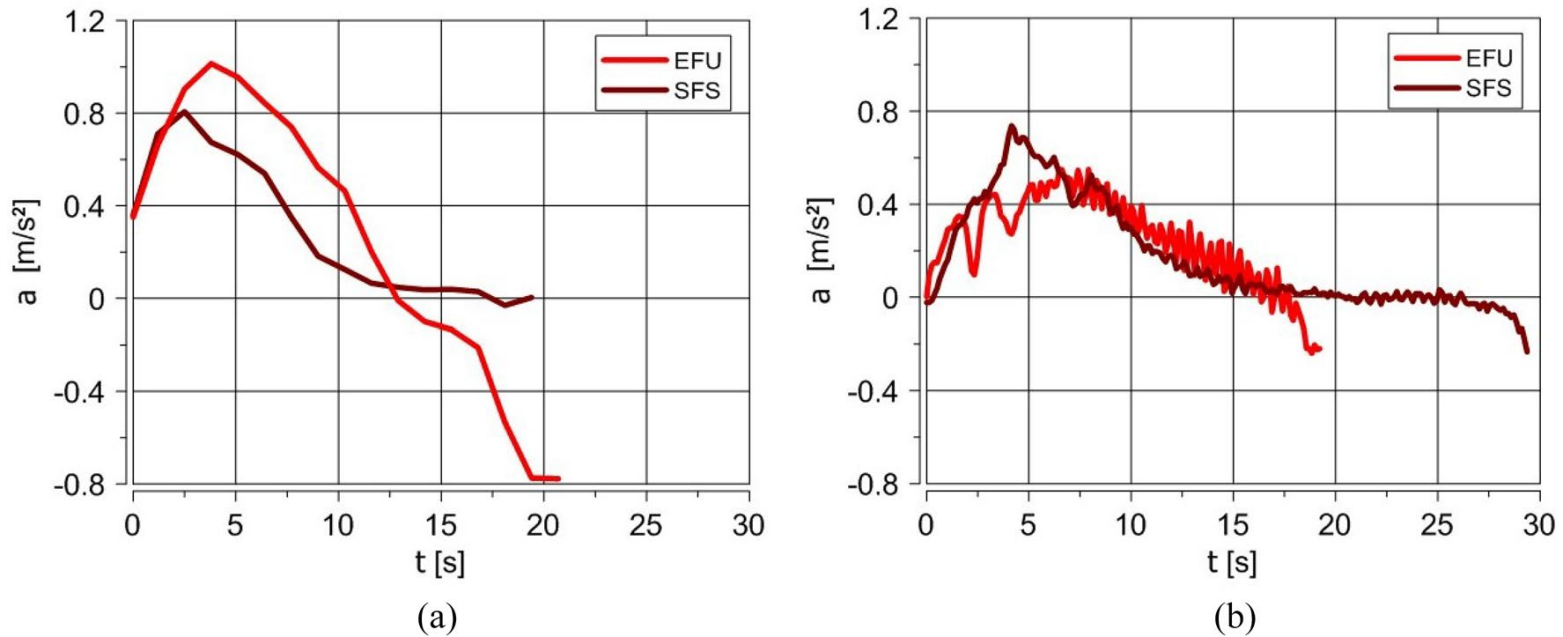
The comparison of truck acceleration for standard and EFU fueling for an empty (**a**) and loaded truck (**b**) (EFU—Emergency Fueling Unit, SFS—Standard Fuel System). (Source: Authors).

In the beginning, the engine was started using EFU (the test was repeated five times, with 100% effectiveness). Then, the acceleration tests were carried out. The comparison of averaged (each test was repeated three times) truck velocity and its acceleration for empty and then for loaded truck is shown in Figs. 7 and 8.

## Conclusions

The EFU allowed for the effective start of the engine and the drive on 5 km distance with at least 60% of the permissible load. The later tests showed that the distance could be much longer. The truck drove 30 km/h. The velocity was limited by the engine speed for the third gear engaged during the tests. Generally, the test results show good repeatability of the measured parameters. The different course of truck velocity diagrams presented in Fig. 7 for standard and EFU fueling resulted from a completely different engine fueling method and other physicochemical properties of the fuel mixture used in EFU. For EFU fueling, the absence of any standard control elements, including the engine speed limiter, influenced the obtained results (higher truck speed). The empty truck fuelled with EFU achieved a higher maximum acceleration value. It was 0.9 m/s^2^ compared to 0.55 m/s^2^ for the standard fuel system. The truck's tests confirmed that it is possible to start, accelerate, and drive the empty and loaded truck without any electrical installation, which could happen when the vehicle is affected by EMP.

The EFU presented in the paper is a prototype. It is possible to manufacture the set dedicated to any CI engine with an air compressor. The different dimensions of engines will not be a serious problem. Adapting the EMP to a specific engine requires different lengths and shapes of air and fuel lines and unique regulation of the air and fuel flow parameters. Also, the different share of rapeseed oil in the blend would be recommended for each particular engine type. It depends on the engine compression ratio (the higher CR, the more rapeseed oil in the blend).

## Summary

Coronal mass ejections (CMEs) strong enough to create electromagnetic effects at latitudes below the auroral oval are frequent events that could soon substantially impact electrical grids and other systems, including transportation^36^. Several nations are building powerful nuclear bombs designed to produce super-electromagnetic pulse (EMP) waves capable of devastating all electronics from computers to electric grids for hundreds of miles. The potential threat of using destructive weapons for local electric systems or specific targets like a part of the transportation system is difficult to estimate. Still, there is a widely held belief in the imminent probability of mass destruction weapons being used by terrorists against civilian targets. The author of^37^ wrote: “Once the disaster has been mitigated, steps are implemented to bring back capacity with existing infrastructure. If a mode has been impaired, the usage of alternative modes and infrastructure has to be considered. The goal is to maintain operational as many elements of the transport system as possible”.

The paper presents the Emergency Fueling Unit prototype that enables running a vehicle affected by EMP of high energy when the standard protection systems like insulations or protection shields are not efficient, and the electric vehicle system is destroyed. In case of natural disaster or deliberate use of EMP to destroy infrastructure, the EFU allows transportation of people and goods on a short distance despite damages caused by EMP in a vehicle. The unit even could protect human life, enabling the safe rescue from the threatened area. The presented unit is a proposal of an electric-independent, add-on system that could be developed and applied in emergency vehicles powered by IC engines with pneumatic installation. The solution is cheap and simple, but it is effective. It can be adjusted to practically any construction of CI engine (the engine must be equipped with an air-compressor) as an assembly package.

In the paper, we do not mention the threats connected with electric vehicles application and their network control in the context of EPM. However, the effects of an EMP on electric vehicles have yet to be studied and is currently unknown. The discussion on this subject is crucial for our safety and the development of transport. When developing our more sophisticated transport means, we should find solutions to protect us and ensure our security. The paper's content should reflect how our civilisation, especially transportation, depends on electricity and how we forget that the threat is not an abstraction.
